# Peri‐Implant Subcapital Femur Fracture Post Cephalomedullary Nail Fixation

**DOI:** 10.1155/cro/4761266

**Published:** 2026-09-02

**Authors:** Oisin O′Connor, Orla Hennessy, Brendan O′Daly

**Affiliations:** ^1^ Royal College of Surgeons Ireland, Dublin, Ireland; ^2^ Department of Trauma and Orthopaedic surgery, Tallaght University Hospital, Dublin, Ireland

**Keywords:** case report, cephalomedullary nail, hip fracture, peri-implant

## Abstract

**Presentation:** A 65‐year‐old female with a prior right‐sided cephalomedullary nail fixation for a pertrochanteric neck of femur fracture fell from a gate onto her right hip. She was unable to weight‐bear on her right leg and presented to the emergency department.

**Diagnosis:** X‐ray imaging in the emergency department diagnosed a peri‐implant subcapital neck of femur fracture at the base of the lag screw threads.

**Treatment:** After evaluation of multiple treatment options, the patient was managed conservatively with physiotherapy, progressive weight‐bearing and bone protection interventions.

**Discussion:** An unusual case of a peri‐implant femoral neck fracture that is not well described by current classification systems is presented. This case presents interesting questions in the management of a rare complication of cephalomedullary nailing.

## 1. Introduction

Neck of femur fractures are a common injury presenting to the emergency department, with an Irish study showing incidence of 389.3 per 100,000 from 2017 to 2019 [1]. Surgical management options are largely divided into those for intracapsular and extracapsular fractures, with cephalomedullary nailing being a common management approach for extracapsular fractures. As global populations age, rates of proximal femoral fractures, and therefore their complications, are projected to rise over the coming decades [2]. While advances in surgical and medical management have improved overall care, proximal femoral fractures continue to cause mortality and morbidity in elderly populations and are associated with significant socioeconomic considerations [3, 4].

Complications after nailing include screw cut‐out, periprosthetic fractures of the metaphyseal or diaphyseal component and nonunion [5–7]. Risk factors for subsequent ipsilateral injuries include female gender, older age, trauma events and osteoporosis [8, 9]. Osteoarthritis has been shown to be a protective factor [10]. Peri‐implant fractures surrounding the lag screw of the cephalomedullary nail are rare occurrences in the literature; only a single previous case was identified upon review, although that case differed due to associated implant failure [11].

In this case report, we present a 65‐year‐old‐female who sustained a peri‐implant fracture surrounding the tip of her cephalomedullary nail lag screw. The purpose of this report is to present this unusual case, discuss its potential management options and future implications for the patient, as well as wider classification issues surrounding peri‐implant fractures.

## 2. Case Report

**Figure 1:**
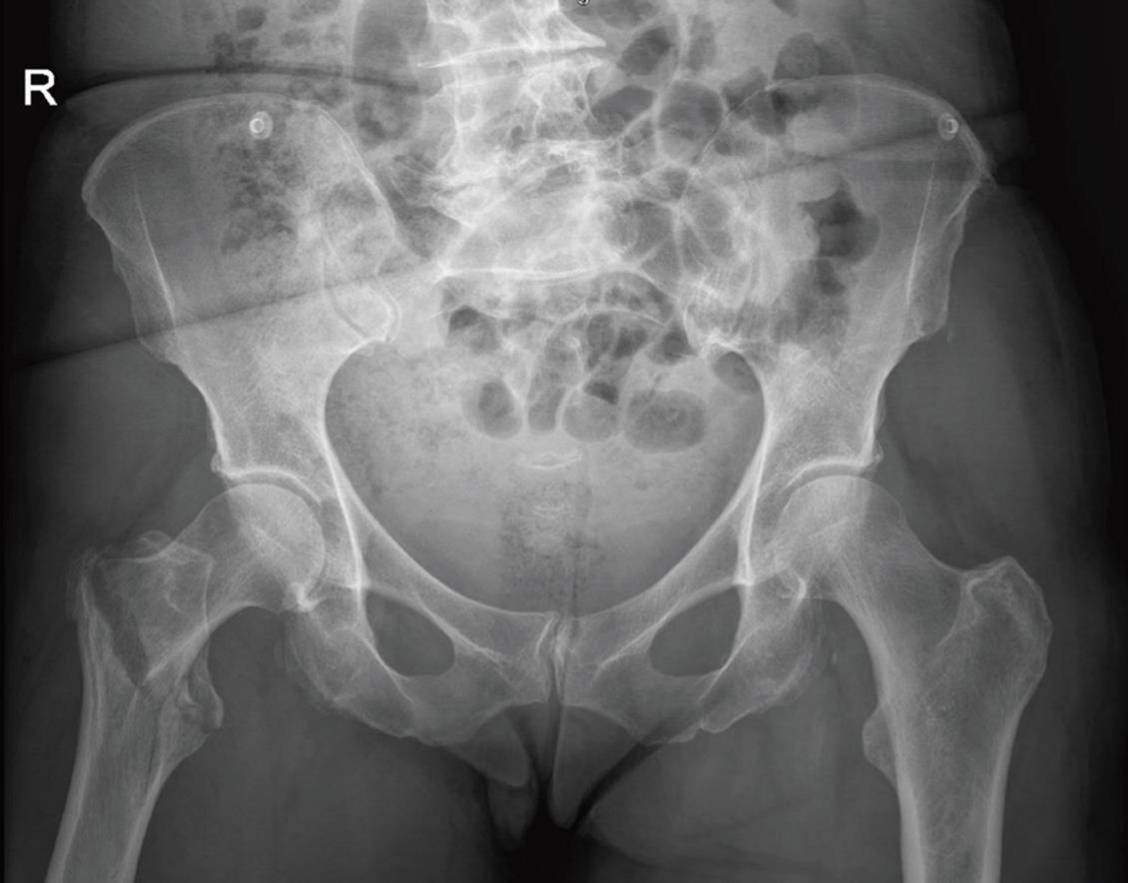
AP pelvic radiograph after initial injury showing right‐sided pertrochanteric hip fracture.

Our patient initially presented aged 57 years following a mechanical fall, having tripped over a wire on the ground and landed on a concrete surface on her right‐hand side. She was brought by ambulance to her local hospital, where pelvic x‐ray imaging confirmed a right‐sided pertrochanteric neck of femur fracture (AO classification 31‐A2), as seen below in Figure 1.

Her background history was significant for a diagnosis of mild hip osteoarthritis and prior bilateral knee arthroscopies. She was a nonsmoker and did not take any regular medications. She was extremely active, working daily in a busy farming environment, and this was a key concern for her recovery.

**Figure 2:**
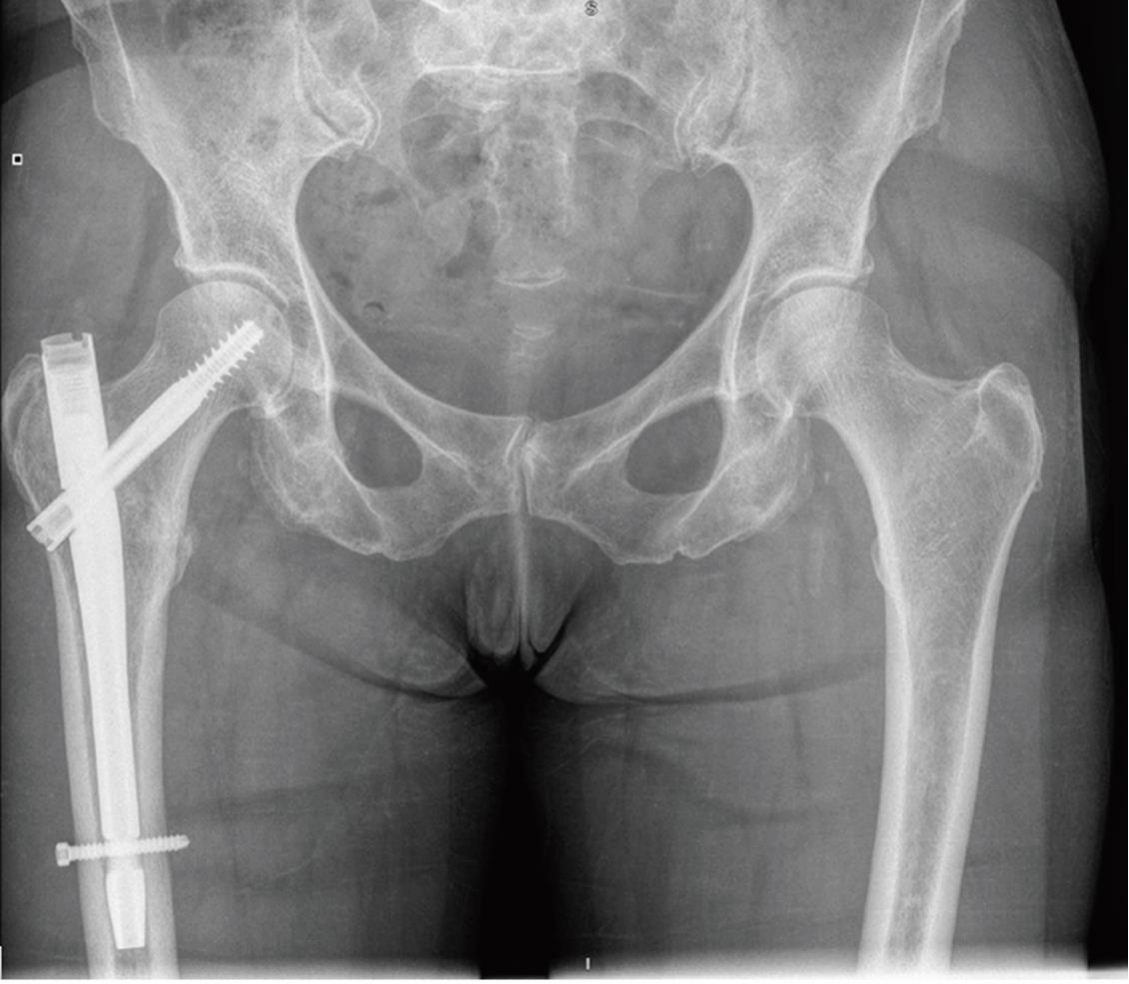
AP pelvic radiograph 6 weeks post‐surgery.

She underwent right‐sided cephalomedullary nail fixation with a 180‐mm Gamma‐3 nail, a 90‐mm lag screw at 130° and a 5 × 40‐mm distal locking screw. Intraoperative screening and postoperative departmental radiographs demonstrated satisfactory fracture fixation and a tip‐apex distance of 20 mm. She mobilised successfully on Day 1 postoperatively and was discharged well 6 days postoperatively. Follow‐up x‐ray imaging at 6 weeks postoperatively showed satisfactory alignment of her intramedullary nail and evidence of fracture healing, as seen below in Figure 2.

**Figure 3:**
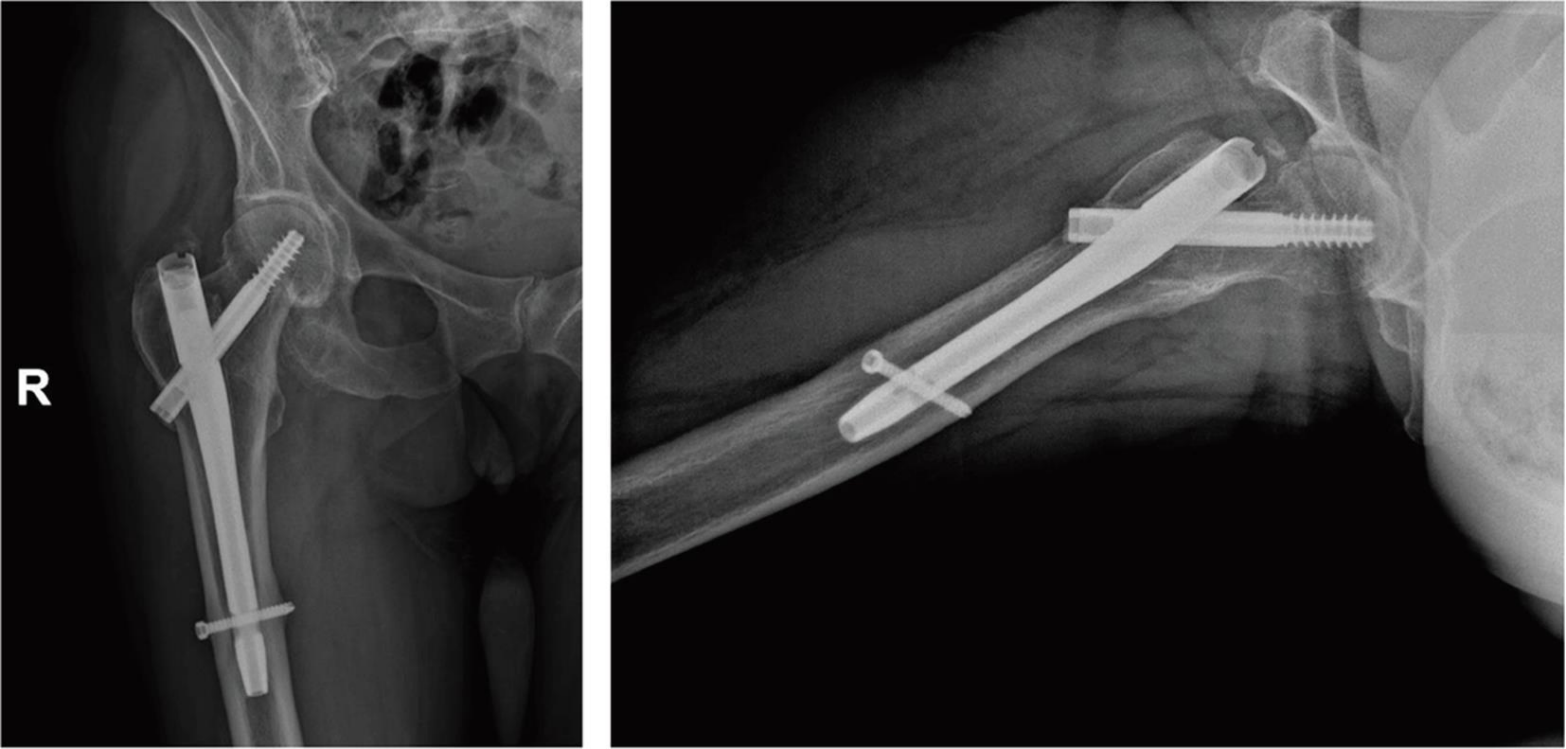
AP and lateral radiographs of right proximal femur after subsequent injury demonstrating right‐sided peri‐implant subcapital neck of femur fracture.

Seven years after this injury, she had a further mechanical fall directly on to her right lateral hip when a gate collapsed beneath her. Following this, she was unable to weight‐bear. Pelvic x‐rays demonstrated a right‐sided subcapital neck of femur fracture at the base of the threads on the lag screw in situ, as seen in Figure 3. On detailed review of her x‐ray imaging, there was evidence of minor lucency surrounding the lag screw of the implant; however, overall implant alignment remained satisfactory. On examination, the patient was weight‐bearing with mild to moderate pain in her right groin; this pain was exacerbated by internal rotation of the hip. She demonstrated a positive Stinchfield test.

**Figure 4:**
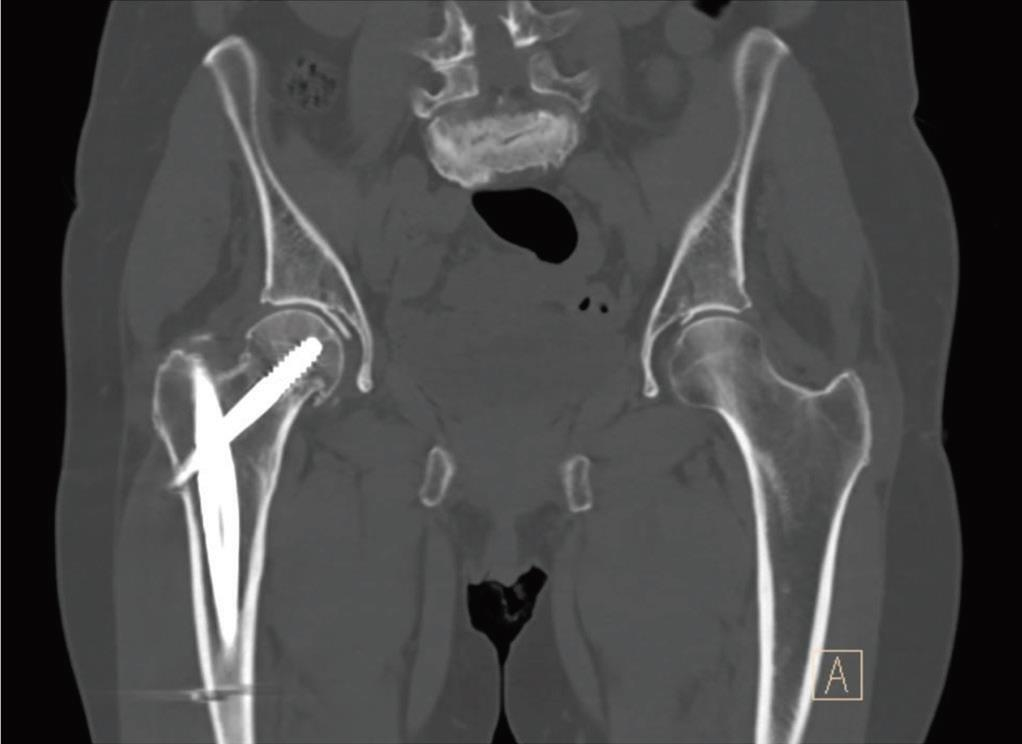
AP pelvis CT slice 9 days post subsequent injury.

**Figure 5:**
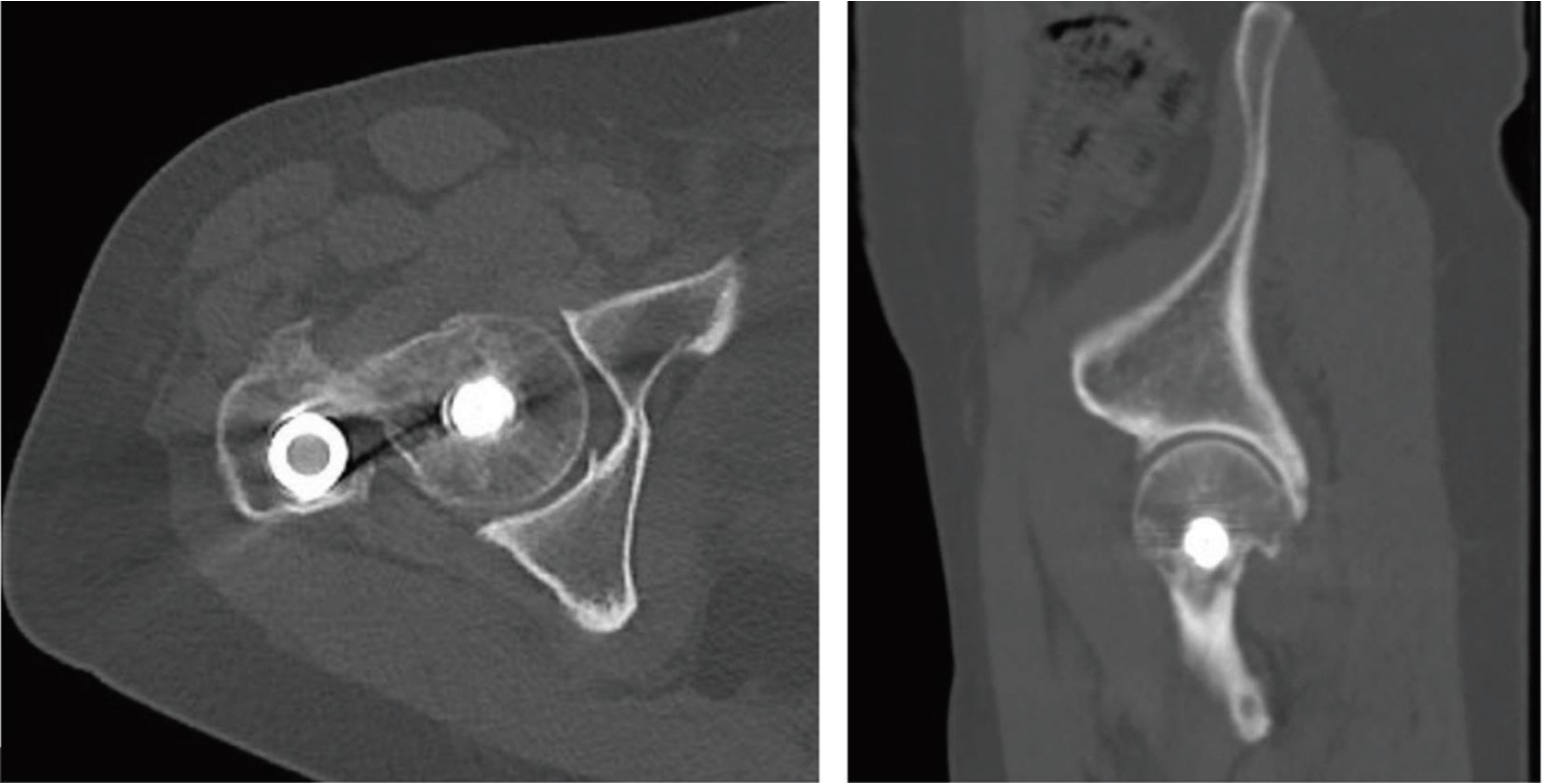
Axial and sagittal CT slices of right hip 9 days post subsequent injury.

Computed tomography (CT) imaging of this subsequent injury was obtained to better assess fracture morphology and implant loosening, as seen in Figures 4 and 5. This demonstrated a complete, minimally displaced subcapital neck of femur fracture surrounding the threads of the lag screw from the intact cephalomedullary nail.

Consideration was given to various management options, including revision fixation, total hip arthroplasty and non‐operative management. Due to the potential morbidity associated with repeat surgical intervention, initial non‐operative management was chosen, comprosing progressive weight‐bearing (initially protected using crutches), physiotherapy and appropriate analgesia.

**Figure 6:**
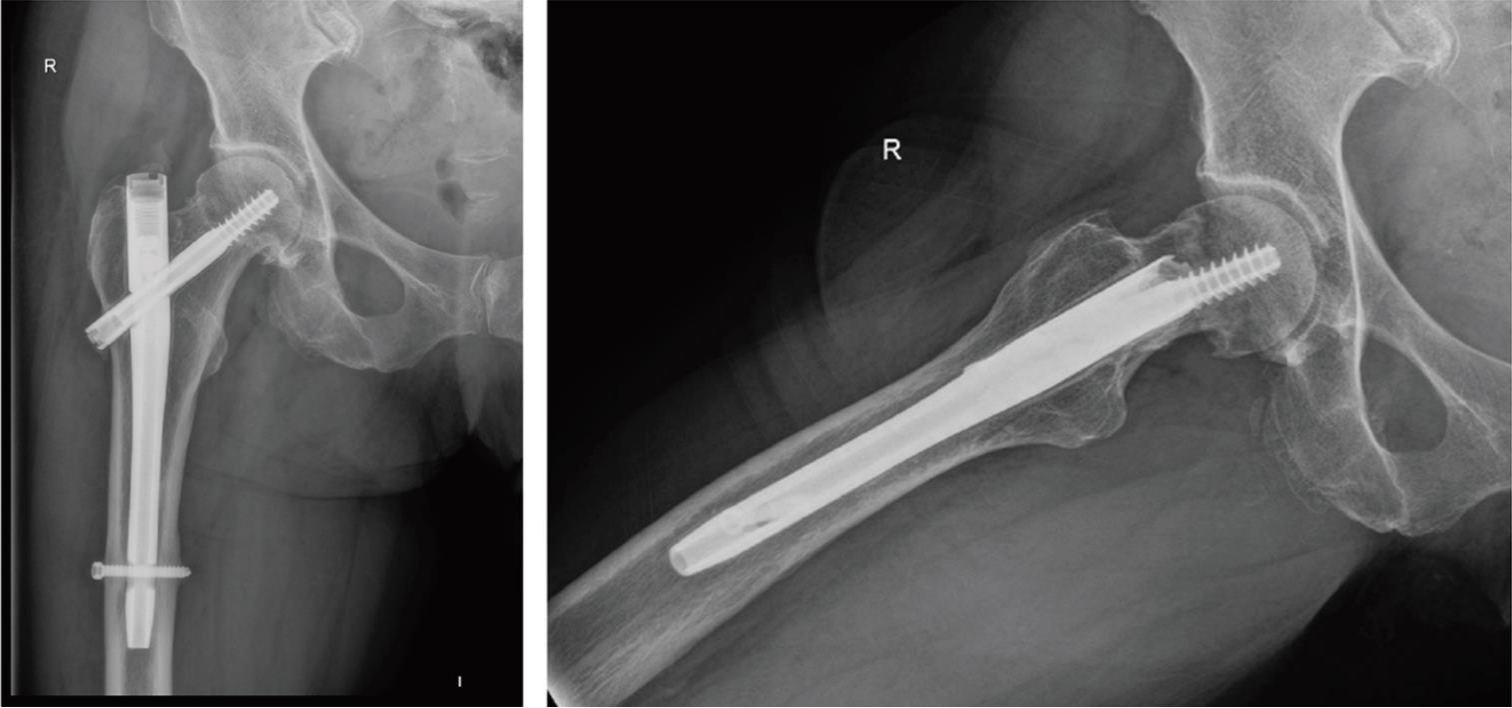
AP and lateral radiographs at 6 weeks following subsequent injury.

**Figure 7:**
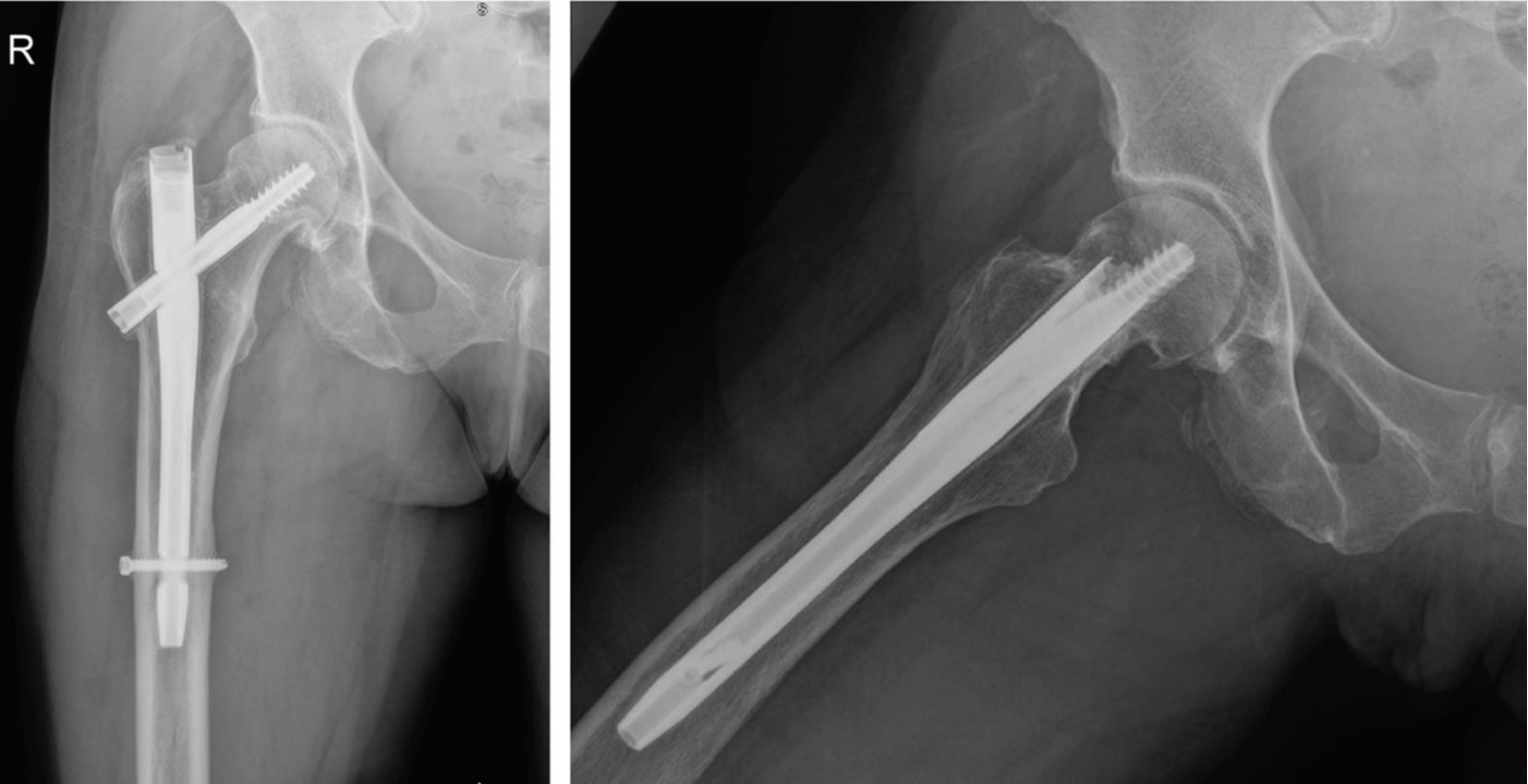
AP and lateral radiographs at 6 months following subsequent injury.

**Figure 8:**
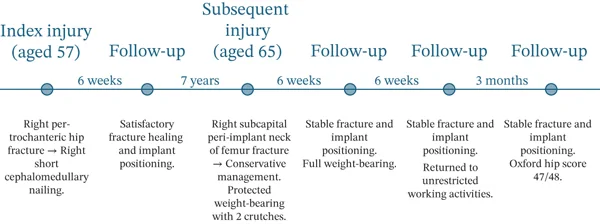
Timeline of patients journey.

The patient recovered well with non‐operative management, returning to full weight‐bearing at 6 weeks and returning to work by 3 months without any limitations in work activities. Groin pain on internal rotation and Stinchfield test resolved by 6 weeks. Follow‐up x‐ray imaging at 6 weeks and 6 months post‐injury demonstrated stable fracture alignment and evidence of bony healing, as seen in Figures 6 and 7. Oxford hip scoring at 6 months post‐injury showed a score of 47 representing satisfactory joint function in activities of daily living. The overall patient journey is represented graphically below in Figure 8.

## 3. Discussion

Peri‐implant fracture incidence post cephalomedullary nailing has been shown to be between 2.1% and 2.4% [7, 12]. The majority of these fractures occur in the femoral shaft, whereas reporting of more proximal fractures surrounding the lag screw is sparse. Indeed, a review of the literature revealed only one case study of an 87‐year‐old patient with a similar fracture pattern to that described in our case [11]. However, this case differed due to the involvement of implant failure, as the distal threaded tip of the lag screw was found to be broken. This patient underwent a cemented dual‐mobility total hip replacement.

This patient had radiographic evidence of pre‐existing, mild degenerative changes in her right hip joint, including medial joint space narrowing and subchondral sclerosis. However, throughout the course of this report, she remained largely asymptomatic in this regard, continuing to carry out her activities of daily living without limitation.

Revision fixation after failure of cephalomedullary nail fixation has been shown to be associated with significant morbidity. A study of reoperation rates after total or hemiarthroplasty for failed trochanteric fracture internal fixation found 16% of patients required further reoperation, with standard‐length femoral stems demonstrating an increased risk of reoperation [13]. A similar study of total hip arthroplasty outcomes after failed proximal femur internal fixation showed a reoperation rate of 15%, an intraoperative fracture rate of 17% and a dislocation rate of 6.8% [14]. The risk of avascular necrosis following undisplaced (Garden Types 1 and 2) intracapsular neck of femur fractures in the current literature is between 3% and 6% [15, 16]. The subsequent fracture suffered by this patient was a Garden Type 2.

The risks and benefits as discussed of each of these alternative treatment approaches, including total hip replacement or revision fixation, were taken into account during this case. Due to the significant morbidity associated with such procedures, a conservative management approach was initially chosen. Of note, as the fracture line traversed the intact in situ lag screw, this was already providing a degree of stability to the fracture site.

This periprosthetic fracture pattern is not well described by any of the current, commonly used classification systems for periprosthetic hip fractures which focus predominantly on fractures after joint arthroplasty. The most commonly used periprosthetic fracture classification system is the Vancouver system, which divides fractures into Groups A (at the level of the trochanters), B (surrounding the stem) and C (below the level of the stem) [17]. These periprosthetic fracture classification systems are not designed to evaluate peri‐implant fractures as a whole and have not been validated for this use.

Classification systems such as the unified classification system, as well other systems for nonarthroplasty fixation have been proposed—such as those by Chan et al. [18] and the Vergilius classification system—have been proposed to encompass a wider range of periprosthetic fractures [19, 20]. These current systems do not adequately accommodate this example of a periprosthetic fracture. Cases such as this highlight a limitation in current periprosthetic fracture classification systems for a system to include fractures of the femoral neck/head. Classification systems allow for a unified methodology of assessing, treating and studying outcomes related to peri‐implant fractures.

This patient achieved a successful recovery following conservative management of her undisplaced peri‐implant fracture. She returned to rigorous farming activities with little to no self‐reported limitations. At the 6‐month follow‐up, the patient stated: ‘whilst hip pain is thankfully rare over the past month of heavy physical workload, I am constantly mindful of not having the same muscle mass on the right side withstand the impact from a sheep.’

Oxford hip score at 6 months postinjury showed satisfactory joint function with a score of 47. X‐ray imaging at 6 months follow‐up demonstrated further interval healing without fractured displacement or sign of avascular necrosis. The patient was referred for a dual‐energy x‐ray absorptiometry (DEXA) bone scan, which was performed 5 months postinjury and revealed a *T*‐score of −2.1, in keeping with a diagnosis of osteopenia.

This case report demonstrates the potential feasibility of nonoperative management in an undisplaced intracapsular peri‐implant neck of femur fracture. Clinicians should thoroughly evaluate the future risks of repeat intervention in peri‐implant fractures versus the risk of future avascular necrosis and early onset arthritis. Of note, this is only a single case report of successful nonoperative management and should be interpreted with caution. Further case reports and longer term outcome data in similar cases are required.

Follow‐up duration is a limitation of this case report, as it was limited to 6 months. Therefore, further evaluation of future joint function was not reported. The patient remains under follow‐up, should her symptoms deteriorate and require surgical intervention.

## 4. Conclusion

This case demonstrates a rare fracture pattern of a peri‐implant femoral fracture around a cephalomedullary nail. There are no guidelines to guide the surgeon in the management of this case, nor is it adequately captured by any of the current periprosthetic fracture classification systems in use. This case demonstrates acceptable outcomes in this scenario with a conservative approach.

## Funding

No funding was received for this manuscript.

## Consent

All the patients allowed personal data processing, and informed consent was obtained from all individual participants included in the study.

## Conflicts of Interest

The authors declare no conflicts of interest.

## Data Availability

The data that support the findings of this study are available on request from the corresponding author. The data are not publicly available due to privacy or ethical restrictions.

## References

[1] Al Azawi L. , Hughes A. J. , and Queally J. M. , National Hip Fracture Incidence-Past, Present and Future Projections, Irish Medical Journal. (2022) 115, no. 1, 35279053. 35279053

[2] Cooper C. , Campion G. , and Melton L. J. , Hip Fractures in the Elderly: A World-Wide Projection, Osteoporosis International. (1992) 2, no. 6, 285–289, 10.1007/BF01623184, 1421796. 1421796

[3] Haleem S. , Choudri M. J. , Kainth G. S. , and Parker M. J. , Mortality Following Hip Fracture: Trends and Geographical Variations Over the Last Sixty Years, Injury. (2023) 54, no. 2, 620–629, 10.1016/j.injury.2022.12.008, 36549980. 36549980

[4] Ferris H. , Brent L. , and Sorensen J. , Cost of Hospitalisation for Hip Fracture-Findings From the Irish Hip Fracture Database, Osteoporosis International. (2022) 33, no. 5, 1057–1065, 10.1007/s00198-021-06294-7, 35015086. 35015086 PMC8749353

[5] Erez O. and Dougherty P. J. , Early Complications Associated With Cephalomedullary Nail for Intertrochanteric Hip Fractures, Journal of Trauma and Acute Care Surgery. (2012) 72, no. 2, E101–E105, 10.1097/TA.0b013e31821c2ef2, 22439243. 22439243

[6] Marti-Garin D. , Fillat-Goma F. , Marcano-Fernandez F. A. , Balaguer-Castro M. , Murias Alvarez J. , Pellejero R. , Fernández J. S. , Torner P. , and Vives J. M. , Complications of Standard Versus Long Cephalomedullary Nails in the Treatment of Unstable Extracapsular Proximal Femoral Fractures: A Randomized Controlled Trial, Injury. (2023) 54, no. 2, 661–668, 10.1016/j.injury.2022.11.037, 36411103. 36411103

[7] Larose G. , Tufescu T. , and Graham C. , Periprosthetic Fracture Rate After Short and Long Hip Nails: Analysis of a Regional Health Database, Injury. (2022) 53, no. 6, 2195–2198, 10.1016/j.injury.2022.03.001, 35341598. 35341598

[8] Cook R. E. , Jenkins P. J. , Walmsley P. J. , Patton J. T. , and Robinson C. M. , Risk Factors for Periprosthetic Fractures of the Hip: A Survivorship Analysis, Clinical Orthopaedics and Related Research. (2008) 466, no. 7, 1652–1656, 10.1007/s11999-008-0289-1, 18470576. 18470576 PMC2505237

[9] Chen X. and Yuan Z. , Risk Factors and Risk-Stratified Prevention of Periprosthetic Fractures After Hip Arthroplasty in Patients Aged >/=80 Years: A Retrospective Cohort Study With Prospective Validation, Medicine. (2026) 105, no. 6, e47521, 10.1097/MD.0000000000047521, 41650033. 41650033 PMC12885698

[10] Cui E. , Durbin J. W. , Zhao A. , Fealy A. , Parel P. M. , Ranson R. , Quan T. , Gill S. , Agarwal A. , Gomez G. , Rao S. , and Thakkar S. C. , Risk Factors for Periprosthetic Fractures Following Total Hip Arthroplasty in Patients Younger Than 50 Years, Journal of Arthroplasty.(2026) 41, no. 4, 1235–1240, 10.1016/j.arth.2025.08.069, 40902689. 40902689

[11] Kostretzis L. , Chatzivasiliadis M. , Tsakiri V. , Konstantinou P. , Ditsiou A. Z. , Kapetanakis S. , and Ditsios K. , Subcapital Femoral Fracture With Cephalomedullary Nail Lag Screw Failure After Healed Intertrochanteric Fracture: A Case Report, Orthopedic Reviews. (2025) 17, 145054, 10.52965/001c.145054. 41069859 PMC12507474

[12] Yamanaka T. , Matsumura T. , Ae R. , Hiyama S. , and Takeshita K. , Risk of Peri-Implant Femoral Fracture After Cephalomedullary Nailing in Older Patients With Trochanteric Fractures, Injury. (2024) 55, no. 6, 111206, 10.1016/j.injury.2023.111206, 37996270. 37996270

[13] Enocson A. , Mattisson L. , Ottosson C. , and Lapidus L. J. , Hip Arthroplasty After Failed Fixation of Trochanteric and Subtrochanteric Fractures, Acta orthopaedica. (2012) 83, no. 5, 493–498, 10.3109/17453674.2012.688724, 22574819. 22574819 PMC3488176

[14] Morice A. , Ducellier F. , Bizot P. , and Orthopaedics and Traumatology Society of Western France (SOO) , Total Hip Arthroplasty After Failed Fixation of a Proximal Femur Fracture: Analysis of 59 Cases of Intra- and Extra-Capsular Fractures, Orthopaedics & Traumatology: Surgery & Research. (2018) 104, no. 5, 681–686, 10.1016/j.otsr.2018.04.015, 29908356. 29908356

[15] Benady A. , Yehiel N. , Efrima B. , Abdellatif A. , Vidra M. , Khashan M. , Ben-Tov T. , and Khoury A. , Risk Assessment of Femoral Head AVN Following Reduction and Fixation of Intracapsular Hip Fractures in Patients Younger Than 65 Years Over a 10 Year Follow Up, Scientific Reports. (2025) 15, no. 1, 10.1038/s41598-025-89974-2. PMC11832920 39962325

[16] Konarski W. , Pobozy T. , Kotela A. , Sliwczynski A. , Kotela I. , Hordowicz M. , and Krakowiak J. , The Risk of Avascular Necrosis Following the Stabilization of Femoral Neck Fractures: A Systematic Review and Meta-Analysis, International Journal of Environmental Research And Public Health. (2022) 19, no. 16, 10050, 10.3390/ijerph191610050, 36011686. 36011686 PMC9408780

[17] Gaski G. E. and Scully S. P. , In Brief: Classifications in Brief: Vancouver Classification of Postoperative Periprosthetic Femur Fractures, Clinical Orthopaedics and Related Research®. (2011) 469, no. 5, 1507–1510, 10.1007/s11999-010-1532-0, 20809166. 20809166 PMC3069264

[18] Chan L. W. M. , Gardner A. W. , Wong M. K. , Chua K. , Kwek E. B. K. , and Singapore Orthopaedic Research Collaborativ E , Non-Prosthetic Peri-Implant Fractures: Classification, Management and Outcomes, Archives of Orthopaedic And Trauma Surgery. (2018) 138, no. 6, 791–802, 10.1007/s00402-018-2905-1. 29532152

[19] Toro G. , Moretti A. , Ambrosio D. , Pezzella R. , De Cicco A. , Landi G. , Tammaro N. , Florio P. , Cecere A. B. , Braile A. , and Medici A. , Fractures Around Trochanteric Nails: The “Vergilius Classification System”, Advances in Orthopedics. (2021) 2021, no. 1, 7532583, 10.1155/2021/7532583. 33520318 PMC7817309

[20] Van der Merwe J. M. , Haddad F. S. , and Duncan C. P. , Field Testing the Unified Classification System for Periprosthetic Fractures of the Femur, Tibia and Patella in Association With Knee Replacement: An International Collaboration, Bone & Joint Journal. (2014) 96, no. 12, 1669–1673. 25452371 10.1302/0301-620X.96B12.34103

